# Do help‐seeking adolescents report more psychotic‐like experiences than young adults on the 16‐item version of the prodromal questionnaire (PQ‐16)?

**DOI:** 10.1111/eip.13597

**Published:** 2024-08-02

**Authors:** Yvonne de Jong, Albert E. Boon, Cornelis L. Mulder, Mark van der Gaag

**Affiliations:** ^1^ Youz Child and Adolescent Psychiatry Parnassia Psychiatric Institute Rotterdam and The Hague The Netherlands; ^2^ Department of Psychiatry, Epidemiological and Psychiatric Research institute Erasmus MC Rotterdam The Netherlands; ^3^ LUMC Curium—Child and Adolescent Psychiatry Leiden University Medical Center Leiden The Netherlands; ^4^ Department of Clinical Psychology Vrije Universiteit Amsterdam The Netherlands

**Keywords:** adolescents, early detection, prodromal questionnaire, psychotic disorder, psychotic‐like experiences, young adult

## Abstract

**Aim:**

To compare psychotic‐like experiences (PLEs) in adolescents and young adults referred to the Mental Health Services (MHSs).

**Methods:**

Participants scored the 16‐item Prodromal Questionnaire (PQ‐16) as part of the intake procedure. Data on the Diagnostic and Statistical Manual of Mental Disorders (DSM) classification and demographic data were collected.

**Results:**

The PQ‐16 was completed by 13 783 respondents (mean age 24.63 years, SD = 6.09; 62.6% female). Overall, the scores on the PQ‐16 were not higher for adolescents (11–17 years; *m* = 4.84, SD = 3.62) than for young adults (18–35 years; *m* = 5.47, SD = 3.85). On PQ‐16 item level, adolescents reported seeing and hearing things more than adults did. Across all age groups, males scored lower on the PQ‐16 than females. Specifically, adolescent males scored lower than other participants. For adolescents and young adults alike, PQ‐16 scores were higher for participants with borderline personality disorder, PTSD, and mood disorder than for those with other DSM classifications.

**Conclusions:**

Although help‐seeking adolescents did not score higher on the PQ‐16 than help‐seeking young adults, more of them reported perceptual anomalies. Irrespective of age, participants with borderline personality disorder, PTSD and mood disorder scored higher on the PQ‐16 than those with other DSM classifications.

## 1 INTRODUCTION

Although the incidence of psychotic disorders is lower in mental‐help‐seeking adolescents (age 12–17) than in help‐seeking adults (18 years and older) (Kessler et al., 2007), these adolescents are more likely to experience psychotic‐like experiences (PLEs, that is perceptual anomalies, unusual beliefs and distorted thinking) (Kelleher et al., 2011). And although PLE prevalence rates differ according to the PLE definition chosen and the questionnaire used (Hinterbuchinger & Mossaheb, 2021; Lee et al., 2016), most researchers agree that adolescents do indeed experience more perceptual anomalies than adults (Linscott & van Os, 2013; Maijer et al., 2019; Schultze‐Lutter et al., 2017).

PLEs are common in the general population, where prevalence rates of auditory perceptual anomalies were found in 12.4% in adolescents and 5.8% in adults (Maijer et al., 2018). The prevalence of visual and auditive anomalies together was 7% in adolescents aged 13–16 (Kelleher, Connor, et al., 2012) versus 1.7%–5% for adults 18 years and older (Scott et al., 2008; Yates et al., 2021). Little research has been done on the phenomenon of PLEs in addition to the concept of Clinical High Risk for psychosis (CHR‐p)  in help‐seeking youth and young adults, which is surprising given that psychosis often starts at this age (McGrath et al., 2016). Two studies found more perceptual anomalies in help‐seeking young adolescents (11–15 years old) than in older adolescents and adults (16–40 years old) (Brandizzi et al., 2014; Schultze‐Lutter et al., 2017).

Although PLEs in adolescents are strong predictors for a later psychotic disorder (Healy et al., 2019), the use of instruments to screen for psychosis during adolescence (Ising et al., 2012) may generate more false positives than screening in adult populations (Savill et al., 2018; Schimmelmann et al., 2007; Schultze‐Lutter et al., 2017), two possible consequences are stigmatization and unnecessary treatment (Ziermans et al., 2011). And although the screening and treatment of PLEs in Mental Health Services (MHSs) has been shown to be effective in preventing psychosis (Fusar‐Poli et al., 2015; McGorry & Mei, 2018; van der Gaag et al., 2019), early‐stage PLEs are seldom detected during regular MHS treatment (Schimmelmann et al., 2013; Stentebjerg‐Olesen et al., 2016).

The need for a structured screening approach is clear (Schimmelmann et al., 2013). One common first‐step screener in the early detection of psychosis is the 16‐item version of the Prodromal Questionnaire (PQ‐16; Ising et al., 2012), which is easy to use in MHS and can signal an increased risk of psychotic disorders at an early stage (de Jong et al., 2018; Ising et al., 2012; Pelizza et al., 2019). Relative to standard referral methods (Rietdijk et al., 2012), its use in adults as a first‐step screener in MHS resulted in a 3‐fold higher detection of clinical high risk state for psychosis (CHR‐p; Fusar‐Poli, 2017).

Interestingly, higher cut‐off points on the PQ‐16 were required to detect psychosis in help‐seeking adolescents than those required to detect psychosis in help‐seeking adults (de Jong et al., 2020). And in both the general and the help‐seeking populations, females of all ages experienced more PLEs than males (Fonseca‐Pedrero et al., 2008; Maric et al., 2003; Scott et al., 2008; Verdoux & van Os, 2002; Wigman, Vollebergh, et al., 2011). PLEs are in general a transdiagnostic phenomenon overlapping with general psychopathology (Pain et al., 2018; Stentebjerg‐Olesen et al., 2016) and are associated with several diagnostic and statistical manual of mental disorders (DSM) classifications, especially depression, post‐traumatic stress disorder and borderline personality disorder (D'Agostino et al., 2019; Fusar‐Poli et al., 2013; Fusar‐Poli et al., 2014; Pain et al., 2018; Stentebjerg‐Olesen et al., 2016). Further research into PQ‐16 scores by age, gender and DSM‐classification can contribute to the alertness of healthcare professionals to PLEs when they work in departments that focus on specific disorders, ages or females and can contribute to a better detection method of psychoses.

To our knowledge, no comparisons have been published of PQ‐16 scores in mental‐help‐seeking populations of different age, gender and DSM diagnostic groups. To investigate whether it is important to further explore gender and age in the interpretation of PQ‐16 scores and of the future of cut‐off scores in the screening process of psychosis, we therefore described and compared age‐ and gender‐stratified PQ‐16 total scores in adolescents and adults who had been referred to mental health services in the Netherlands. We also determined mean scores in different DSM‐classification groups, hypothesizing that (1) more adolescents than young adults would score above the cut‐off score of the PQ‐16; (2) adolescents would score higher than adults on the PQ‐16 total score; (3) females would have higher PQ‐16 total scores than males; (4) participants with depression, post‐traumatic stress disorder and borderline personality disorder would have higher PQ‐16 total scores than those with other DSM diagnoses, and (5) adolescents would score higher than adults on visual and auditive anomalies, that is seeing and hearing things (items 8 and 13). In hypothesis 4, we excluded psychotic and bipolar disorders. This is first because the number of these diagnoses in our sample are too small to make a reliable statement. Second, it is expected that adolescents with psychotic disorders and bipolar disorders with a manic component present, will experience various PLEs and therefore will score high at the PQ‐16. The PQ‐16 consists of various PLEs that are based on the items of the comprehensive assessment of at‐risk mental states (CAARMS) that determines an at‐risk mental state (ARMS) or psychotic threshold.

## 2 METHODS

### 2.1 Setting and procedures

The study took place in the Netherlands, where help‐seeking adolescents and young adults aged 11–35 were referred between January 2007 and March 2017 by general practitioners and services and attended either a Child and Adolescent Mental Health Service (CAMHS) in Rotterdam, Hoogvliet, Spijkenisse, Den Haag, Voorburg, Zoetermeer or a Centre for Adult Mental Health Service (AMHS) in Den Haag, Zoetermeer or Delft, that provided specialist assessment and treatment for psychiatric disorders. Only participants with a psychiatric classification (DSM‐IV) were eligible for the study. Participants with only V‐codes (other non‐diagnosable conditions that may be a focus of clinical attention) or adjustment disorders were referred elsewhere. The PQ‐16 was a necessary part of the intake procedure, and the scores were available to the clinicians involved in the participants' treatment.

### 2.2 Measures

The PQ‐16 (Ising et al., 2012) is a first‐step psychosis‐risk screener, which, in the Netherlands, is usually followed by an interview with the CAARMS (Yung et al., 2005) to determine a CHR‐p (Fusar‐Poli, 2017) or whether the psychosis threshold had been met. The PQ‐16 assesses the presence of nine hallucination‐like items (items 3, 4, 6, 8, 9, 12, 13, 15 and 16); five delusion‐like items (items 2, 5, 10, 11 and 14); and two subclinical negative‐symptom items (items 1 and 7) on a two‐point scale (true/false). It was empirically constructed on the basis of each item's independent contribution to the UHR status on the CAARMS. Although a cut‐off of 6 or more items agreed was usually applied in help‐seeking adolescents and adults (Ising et al., 2012; Savill et al., 2018), we have previously shown that a cut‐off of ≥7 items agreed was more appropriate for adolescents aged 12–17 who were seeking help (de Jong et al., 2020). We applied both cut‐offs in this sample. The DSM IV classification and the global assessment of functioning (GAF; Endicott et al., 1976) score were determined by a licenced psychologist or psychiatrist on the basis of a clinical interview during the intake procedure. In addition, demographic data were collected.

### 2.3 Statistical analyses

For comparison with work by other researchers, we divided the sample into participants aged 17 years and younger (18−) and those 18 years and older (18+). To determine prevalence rates per item agreed on the PQ‐16 and cut‐off percentages (≥6 and ≥7), we calculated descriptive statistics. Independent sample *t*‐tests were used to examine the difference in mean total PQ‐16 score between the 18+ and 18− samples. Differences in the percentages reaching the cut‐off in the samples 18+ and 18− were calculated by chi‐square. To determine differences for age and gender in the total PQ‐16 score, we used a factorial ANOVA with two fixed factors: ‘gender’ and ‘belonging to the 18− or 18+ group’. Thereafter we used descriptive statistics to show the PQ‐16 scores per age, differentiated for gender with a confidence interval (CI) of 95%.

Due to differences in sample size and variance, we used the following to determine whether the PQ‐16 total scores were higher for participants with depression, post‐traumatic stress disorder and borderline personality disorder: an ANOVA, followed by a Welch test, followed by Games Howell post‐hoc tests. In this analysis, bipolar disorders were included among the mood disorders and psychotic disorders among the other disorders. For the other analyses, Bonferroni post‐hoc tests were used to correct for multiple testing; and Cohen's d, Eta‐squared, Partial Eta squared and Phi were used to determine effect sizes. Analyses were conducted using SPSS 27.0 (SPSS, 2020).

## 3 RESULTS

### 3.1 Sample characteristics

**FIGURE 1:**
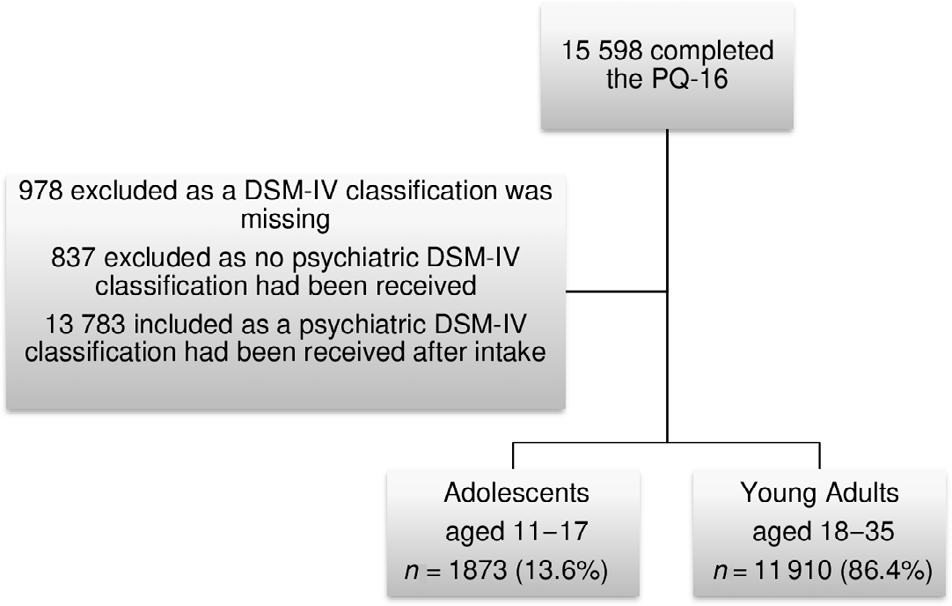
Flowchart participants.

The total sample included 13 783 participants, half of whom (49.7%) were native Dutch, with a mean age of 24.63 years (SD = 6.09, range 11–35). Adults (86.4%) and females (62.6%) were overrepresented in the sample. Figure 1 shows a flowchart of participants. Table 1 compares the characteristics of adults aged 18 years and older with those of adolescents of 17 years or younger.

**TABLE 1:** Sample characteristics per setting, with sample size (*N*), mean (*M*) and standard deviation (SD).

Characteristic	Adults 18+	Adolescents 18−
Age total	*M* (SD)	*M* (SD)
	26.2 (4.9)	14.5 (1.8)
Gender	*N* (%)	*N* (%)
Male	4335 (36.4)	819 (43.7)
Female	7575 (63.6)	1054 (56.3)
Citizenship	*N* (%)	*N* (%)
Dutch native	5993 (50.3)	852 (45.5)
Migrant background	2644 (22.2)	435 (23.2)
Missing	3273 (27.5)	586 (31.3)
DSM classification	*N* (%)	*N* (%)
Autism	56 (0.5)	192 (10.3)
ADHD^a^	3350 (28.1)	442 (23.6)
Mood	2264 (19.0)	317 (16.9)
Bipolar disorder	121 (1.0)	6 (0.3)
Anxiety	2177 (18.3)	216 (11.5)
Behavioural	106 (0.9)	103 (5.5)
PTSD^b^	945 (7.9)	111 (5.9)
Borderline	387 (3.2)	18 (1.0)
Pers. NAO^c^	843 (7.1)	20 (1.1)
Eating disorder	697 (5.9)	53 (2.8)
Psychosomatic	343 (2.9)	41 (2.2)
Psychotic disorder	35 (0.3)	17 (0.9)
Gender	238 (2.0)	1 (0.1)
Disorder of adolescence NAO/mental disorder NAO^d^	10 (0.1)	272 (14.5)
Other	338 (2.8)	64 (3.4)
Total	11 910 (100.0)	1873 (100.0)
GAF	*M* (SD)	*M* (SD)
	57.0 (6.6)	52.9 (5.6)

^a^ ADHD, attention deficit and hyperactivity disorder.

^b^ PTSD, post‐traumatic stress disorder.

^c^ Pers. NAO, personality disorder not otherwise specified.

^d^ NAO, not otherwise specified.

#### 3.1.1 Hypothesis 1

With the cut‐off set to 6 or more items agreed, more adults than adolescents met the cut‐off (42.7% vs. 37.4%, *X* ^2^ (1, *N* = 13 783) = 18.33, *p* < .001, Phi = 0.04).

With the cut‐off set to 7 or more items agreed, more adults than adolescents met the cut‐off (34.2% vs. 28.9%, *X* ^2^ (1, *N* = 13 783) = 19.18, *p* < .001, Phi = 0.04).

#### 3.1.2 Hypothesis 2

The mean total score of the PQ‐16 was lower for 18− (*m* = 4.84 (*n* = 1873, SD = 3.62)) than for 18+ (*m* = 5.47, *n* = 11 910, SD = 3.85, *t* (2582.56) = −6.92, *p* < .001, *d* = −0.16).

#### 3.1.3 Hypothesis 3

**FIGURE 2:**
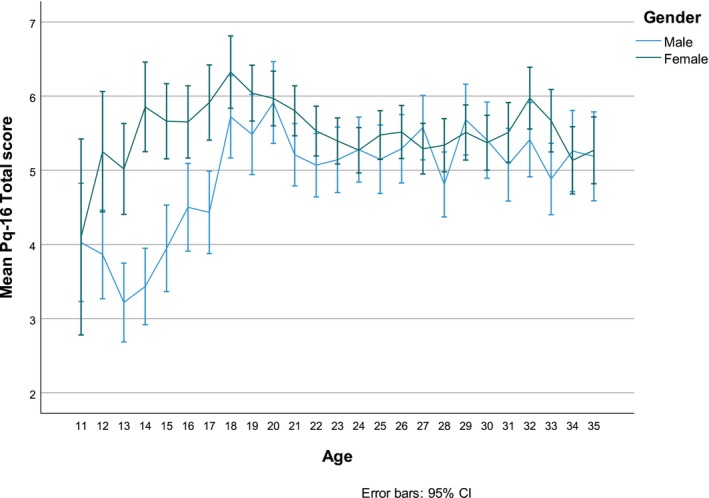
Mean PQ‐16 total score per age, differentiated for gender, with a confidence interval (CI) of 95%.

There was a significant interaction effect between gender and being adolescent or adult (*F* (1, 13 779) = 57.17, *p* < .001, partial eta‐squared = 0.004). Figure 2 shows the total mean PQ‐16 scores per age and gender. While young males had a lower PQ‐16 total score than other participants until the age of 18, young females had a lower PQ‐16 total score than adults until the age of 14.

#### 3.1.4 Hypothesis 4

The PQ‐16 total scores for participants with depression, PTSD, and/or borderline personality disorder (BPD) differed significantly from those with other DSM classifications (*F*(3, 13 779) = 223.,61, *p* < .001, Eta squared = 0.05). This effect persisted after separate tests of the adolescent and adult groups. Post‐hoc analysis showed that adolescents with other DSM classifications (*F* (3, 1869) = 22.82; *n* = 1421, *m* = 4.48, SD = 3.52, *p* < .001) had lower PQ‐16 scores than those with BPD (*n* = 18, *m* = 8.17, SD = 4.45, *p* = .01); those with PTSD (*n* = 111, *m* = 5.60, SD = 3.87, *p* = .02); and those with a mood disorder (*n* = 323, *m* = 5.98, SD = 3.57, *p* < .001). The effect sizes were small (Eta squared = 0.04).

**FIGURE 3:**
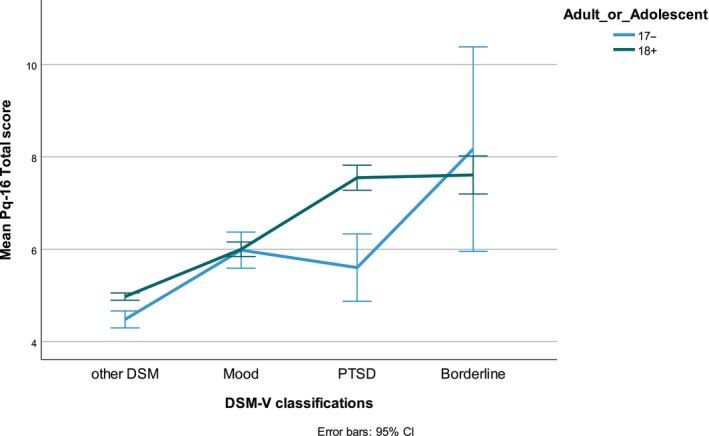
Mean total PQ‐16 score for mood disorder, PTSD and BPD, differentiated for adolescents and adults.

A similar difference was found in adults (*F* (3, 11 906) = 201.64). Adults with other DSM classifications (*n* = 8193, *m* = 4.98, SD = 3.62, *p* < .001) had significantly lower PQ‐16 total scores than adults with BPD (*n* = 387, *m* = 7.61, SD = 4.11, *p* < .001), PTSD (*n* = 945, *m* = 7.55, SD = 4.26) or a mood disorder (*n* = 2385, *m* = 6.00, SD = 3.92, *p* < .001). Effect size was small (Eta squared = 0.05). Figure 3 shows the mean PQ 16‐ total scores for mood disorder, PTSD en BPD, differentiated for adolescents and adults.

#### 3.1.5 Hypothesis 5

One fifth (20.6%) of the 18‐participants reported seeing things versus 16.4% of the 18+ participants (item 8). Hearing things (item 13) was reported by 19.9% versus 12.3%, respectively.

Adolescents scored higher than adults on seeing things (*X* ^2^ (1, *N* = 13 775) = 20.80, *p* < .001, Phi = −0.04) and hearing things (*X* ^2^ (1, *N* = 13 775) = 79.60, *p* < .001, Phi = −0.08).

## 4 DISCUSSION

We compared age‐ and gender‐stratified PQ‐16 scores in adolescents and young adults who had been referred to mental health services in the Netherlands, and found that, contrary to our hypothesis, adolescents did not score higher than young adults. However, adolescents reported more perceptual anomalies than young adults. Unless these experiences occur in combination with other agreed items of the PQ‐16 that reflect other experiences than perceptual anomalies, it may be that they are part of normal neurodevelopment in adolescence (Brandizzi et al., 2014). Besides perceptual anomalies, the PQ‐16 is composed of a combination of delusion‐like PLEs and negative symptom items that predict a CHR‐p status or reaching the psychotic threshold on the CAARMS (Ising et al., 2012). It is also possible that the combination of age with perceptual anomalies is less relevant than the overall combination of any mental disorder or disorders with PLEs. For instance, in adolescents and in adults who report PLEs, any susceptibility to developing psychosis may be increased by the interplay between borderline personality traits and exposure to traumatic events (Sengutta et al., 2019). It is also the case that depression frequently precedes (Fusar‐Poli et al., 2014) and occurs in first‐episode psychosis (FEP) and predicts longer‐term negative outcomes (Upthegrove et al., 2021).

Comparisons for age and gender showed that young females and males scored lower on the PQ‐16 total score than young adults. While young males had a lower PQ‐16 total score than other participants until the age of 18, young females had a lower PQ‐16 total score than adults until the age of 14.

We found a number of similarities with the work of other researchers, one similarity being the higher prevalence of PLEs in females (Ronald et al., 2014; Wigman, van Winkel, et al., 2011). Higher rates of PLEs may be secondary not only to the higher rates of depressive symptoms in females seeking help (Morokuma et al., 2017), but also to the higher rate of trauma in such females (Tolin & Foa, 2006). Although males have an earlier onset of schizophrenia (Miettunen et al., 2019), females may report PLEs at an earlier stage than males because they reach puberty earlier (Fossati et al., 2003; Galdos et al., 1993). Wigman and colleagues also found a higher rate of PLEs in older female adolescents than in younger female adolescents, but this was the case for delusion‐like symptoms and not for hallucination‐like symptoms. They did not find that the rate was increasing in males (Wigman, Vollebergh, et al., 2011).

Although a PQ‐16 total score is based on the answers to fourteen PLEs and two negative symptom items, higher PQ‐16 total scores contain more PLEs. This means that our finding of higher PQ‐16 scores in participants with adult borderline personality disorder and PTSD is in line with other research (Adams & Sanders, 2011) (Slotema et al., 2012) that found a high percentage of PLEs in borderline personality disorder, and a higher association between trauma and PLEs (Tolin & Foa, 2006). Sengutta et al. (2019) also found that borderline features were an important mediator between childhood trauma and PLEs. Another finding that was in line with other research was the higher PQ‐16 scores for mood disorder (Fusar‐Poli et al., 2014; Stentebjerg‐Olesen et al., 2016).

We found that help‐seeking adolescent males scored lower on the PQ‐16 than adolescent females and adults. Although other researchers have also reported on age and gender differences, they did not examine differences between PLEs per age and gender group in help‐seeking adolescents and adults (Brandizzi et al., 2014; Linscott & van Os, 2013; Morokuma et al., 2017; Ronald et al., 2014). However, using the semi structured psychosis‐like symptom interview in a general population of 13–24 years, Sullivan and colleagues did find that the rates of PLEs peaked at around ages 17–19 (PLIKSi; Sullivan et al., 2020).

### 4.1 Implications for clinical practice

It is important to note that not all participants reporting PLEs develop a psychotic disorder or are at risk (Fusar‐Poli, 2017). Between 33% and 66% of the screened population will meet the chosen cut‐off on the PQ‐16 (Azzali et al., 2018; de Jong et al., 2018, 2020; McDonald et al., 2018; Pelizza et al., 2019), which is much higher than the prevalence both of CHR‐p (16%–42% in help‐seeking populations; Lo Cascio et al., 2017; de Jong et al., 2020; Koren et al., 2019) and of true psychotic disorder (2% in help‐seeking adolescents; de Jong et al., 2020). When the cut‐off score is met, the PQ‐16 should thus be followed up by a second‐step interview, which, in the Netherlands, is usually the CAARMS (Yung et al., 2005). Healthcare professionals should remember that PLEs are common in MHS and are reported more by females and less by young males. Our findings suggest that, when screening for psychosis, more staff is needed for screening female and adult samples, because more of them will reach the cut‐off at the PQ‐16. It is also important to take PLEs seriously in disorders, such as depression, PTSD and BPD. This is because the overlap between PLEs and psychopathology is high and the suffering of PLEs or a psychotic development can be missed. Even if there is no transition to psychosis, PLEs are associated with suicidality (Kelleher et al., 2014; Kelleher, Lynch, et al., 2012; Maijer et al., 2019; O'Hare et al., 2021) and help seeking (DeVylder et al., 2014).

### 4.2 Strengths and limitations

A strength is that the study examined PQ‐16 scores in a large sample of adolescents and adults who had a psychiatric DSM classification and had been referred for treatment to an MHS that used the same questionnaire. To our knowledge, this has not been reported before.

This study has the following limitations. First, as we did not assess language or the potential influence of others (such as parents, partners or healthcare professionals) in helping to complete the PQ‐16, we could not determine whether the items had been understood correctly. Second, the prevalence of PLEs was based on the scores on the PQ‐16, which are self‐reported and not supported by a clinical interview. Finally, respondents were selected by intake and psychiatric disorder established by clinicians based on available questionnaires and clinical interviews, and not by a structured clinical interview. Therefore, some diagnoses may have been missed.

## 5 CONCLUSIONS

Although help‐seeking adolescents did not score higher on the PQ‐16 than help‐seeking young adults, more of them reported perceptual anomalies. Irrespective of age, participants with borderline personality disorder, PTSD and mood disorder scored higher on the PQ‐16 than those with other DSM classifications.

## FUNDING INFORMATION

Parnassia Research Academy contributed by granting a stimulation fund for junior investigators of €10 000.

## CONFLICT OF INTEREST STATEMENT

The authors declare no conflict of interest.

## PATIENT CONSENT STATEMENT

The rules of the Dutch Union of Medical‐Ethics Trial Committees for Mental Health Organizations state that written informed consent is not required. Completion of the PQ‐16 was voluntary. Refusing completion of the PQ‐16 had no consequences.

## CLINICAL TRIAL REGISTRATION

Numbers NL.44180.058.13 and NL17123.097.07.

## Data Availability

The data that support the findings of this study are available from the corresponding author upon reasonable request.
